# Rotator Cuff Tears and Mid-Term Shoulder Outcomes after Intramedullary Nail Fixation for Humeral Shaft Fracture: A Minimum Five-year Follow-up Study

**DOI:** 10.5704/MOJ.2411.008

**Published:** 2024-11

**Authors:** R Furuhata, A Tanji, S Nakamura, T Urabe

**Affiliations:** Department of Orthopaedic Surgery, Japanese Red Cross Ashikaga Hospital, Tochigi, Japan

**Keywords:** humeral shaft fracture, intramedullary nail, rotator cuff tear, outcome

## Abstract

**Introduction:**

Antegrade intramedullary nail fixation for humeral shaft fractures yields satisfactory union rates. However, one of the related concerns is damage to the rotator cuff during nail insertion, which may affect long-term outcomes. The effect of a rotator cuff lesion on mid- and long-term shoulder outcomes remains unknown. This study aimed to investigate the incidence of rotator cuff tears 5 years or more after intramedullary nailing for humeral shaft fractures and to determine the impact of post-operative rotator cuff tears on mid-term outcomes.

**Material and Methods:**

We retrospectively identified 27 patients who underwent antegrade intramedullary nail fixation for traumatic humeral shaft fractures and received follow-up for at least 5 years post-operatively. The patients were divided into two groups: those without tears and those with partial or complete tears, diagnosed using ultrasonography. We compared the functional and radiological shoulder outcomes between the two groups.

**Results:**

Of the 27 patients, 10 had partial or complete supraspinatus tears with a mean follow-up of 7.5 years postoperatively. The incidence of acromial spurs was significantly higher in patients with partial or complete tears than in those without tears (P<0.001). There were no significant differences in the age and sex-adjusted Constant score, or the American Shoulder and Elbow Surgeon score between the two groups.

**Conclusion:**

Our results revealed that 37% of patients developed partial or complete supraspinatus tendon tears in the mid-term. Post-operative rotator cuff tears were significantly associated with the formation of acromial spurs; however, they had no significant effect on mid-term shoulder functional outcomes.

## Introduction

Antegrade intramedullary nailing provides solid stability and good load sharing while minimising damage to soft tissues surrounding the fracture site^1^. This surgical procedure yields satisfactory union rates for humeral shaft fractures^2-10^. However, damage to the rotator cuff tendons and articular cartilage during nail insertion can occur. Even if the iatrogenic injury to the rotator cuff tendon is carefully repaired, post-operative rotator cuff re-tears can occur in the short-term^11,12^. Additionally, rotator cuff lesions after intramedullary nail fixation may cause post-operative pain and loss of range of motion^6,8,13,14^. Previous studies examining post-operative outcomes after rotator cuff repair demonstrated that post-operative re-tear was associated with radiographic changes, including the development of glenohumeral osteoarthritis^15-17^ and the reformation of acromial spurs^18^. This raises the concern that rotator cuff tears and articular cartilage injuries due to intramedullary nailing may also cause similar radiographic changes in the long-term. However, the effect of rotator cuff tears following intramedullary nailing on the mid- or long-term shoulder functional and radiological outcomes has not been previously investigated.

This study aimed to investigate the incidence of rotator cuff tears five years or more after intramedullary nail fixation for humeral shaft fractures and to clarify the association of postoperative rotator cuff tears with mid-term shoulder functional and radiological outcomes.

## Materials and Methods

The study protocol was approved by the independent ethics committee of our hospital. All procedures performed in studies involving human participants were in accordance with the ethical standards of the institutional and/or national research committee and with the 1975 Helsinki Declaration and its later amendments or comparable ethical standards.

This study is a retrospective study of patients who underwent osteosynthesis of a humeral shaft fracture at a single general hospital. The inclusion criteria were as follows: (1) patients who underwent antegrade intramedullary nail fixation for humeral shaft fractures between January 2011 and April 2019; (2) acute closed fractures with three weeks or less from injury to surgery; (3) no abnormal findings on preoperative plain radiographs of the shoulder; (4) supraspinatus tendon integrity was confirmed intraoperatively. The exclusion criteria were: (1) loss to follow-up less than five years after surgery; (2) patient refusal to undertake plain radiograph or ultrasound investigations; (3) pathological fracture; and (4) paralysis of the affected upper extremity due to cerebral infarction or other causes.

Five orthopaedic surgeons performed surgeries on patients in the beach-chair position under general anaesthesia. Using a deltoid split approach, the subdeltoid bursa was exposed. The supraspinatus tendon was incised in the direction of the muscle fibers, preserving its insertion at the greater tuberosity. After the introduction of the guide wire, the medullary canal was opened with an awl. We selected the width and size of the intramedullary nail based on the measurement from pre-operative computed tomography and inserted the nail under fluoroscopic guidance. The implants used in this study were MultiLoc humeral nails [DePuy Synthes, Oberdorf, Switzerland], Polarus 2 humeral nails [Acumed, Hillsboro, OR, USA], and Trigen humeral nails [Smith and Nephew, Watfold, UK]. After nail insertion, we inserted the distal and proximal locking screws. The length of the nail and the number of screws depended on the surgeon's judgment. We repaired the supraspinatus tendon with simple interrupted stitches using non-absorbable sutures.

After surgery, the arm was immobilised in a sling for one to two weeks, during which passive range-of-motion training was started; active motion training was started four to six weeks post-operatively.

One examiner with 10 years of experience in shoulder surgery, who was not involved in the surgery, evaluated the shoulder functional outcomes using the Constant score^19^, the American Shoulder and Elbow Surgeon (ASES) score^20^, the Visual Analogue Scale (VAS) score, and range of shoulder motion. The Constant score was adjusted for age and sex^21^. The range of shoulder motion was assessed using a goniometer.

Another examiner, who was blinded to the functional outcome results, evaluated the radiological outcomes. We defined glenohumeral osteoarthritis as Samilson–Prieto grade ≥2, according to a previous study^15^. Acromial spurs were evaluated using a plain radiograph in the scapular-Y view, and the length of a spur was defined as the distance from the point where the inclusion of the anterior edge of the acromion abruptly increased to the tip of the spur^22^. We defined acromial spur formation as a spur ≥5mm^18^. We defined superior migration of the humeral head as an acromiohumeral interval of <6mm^23-25^.

**Fig. 1:**
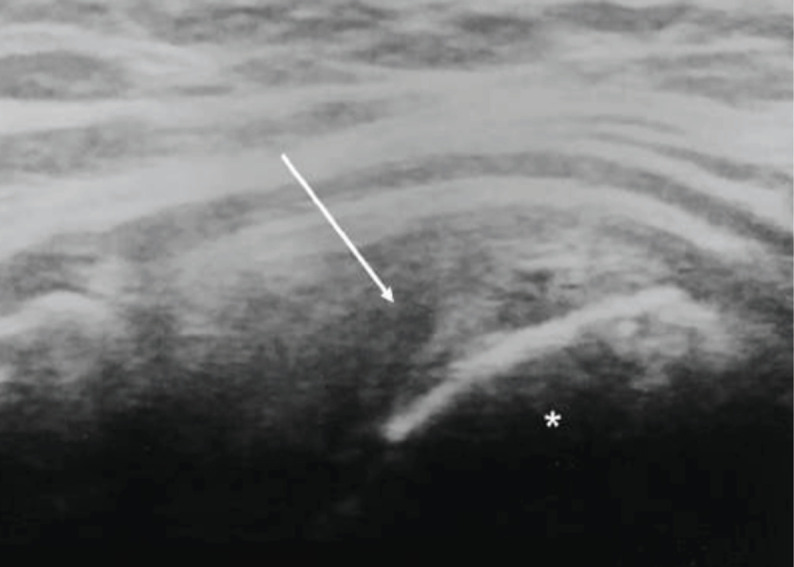
Ultrasonographic evaluation of full-thickness tear of supraspinatus tendon. (Arrow: tendon defect, Asterisk: greater tuberosity).

Rotator cuff integrity was assessed using ultrasonography [Noblus, Hitachi Ltd., Tokyo, Japan] by an orthopaedic surgeon with eight years’ experience in ultrasonographic shoulder examination. According to a previously described procedure^11^, tears were classified as partial or complete (Fig. 1). We compared the functional and radiological outcomes in patients without tears and with partial or complete tears.

We also compared post-operative functional outcomes and the incidence of post-operative rotator cuff tears among the three nails used in this study to determine whether the nail design affected the post-operative outcomes.

All statistical analyses were conducted using SPSS software [version 27.0*, IBM, Armonk, NY, USA]. We used the Mann–Whitney U test to compare the averages of continuous values (age, BMI, time from injury to surgery, time from surgery to final follow-up, adjusted Constant score, ASES shoulder score, VAS, and range of motion). One-way ANOVA with Tukey’s test was used for multiple comparisons. We used Fisher’s exact test to compare the proportions (sex, the affected side of injury, smoking history, diabetes, pre-operative radial nerve injury, position in the shaft, fracture type, glenohumeral osteoarthritis, acromial spur, and superior migration of the humeral head). P<0.05 was considered statistically significant.

## Results

We identified 44 patients who met the inclusion criteria. Of these, 11 patients were excluded from the study due to loss to follow-up (4 died, 4 relocated, and 3 did not attend), 3 due to refusal of radiographic examination, 2 due to pathological fracture, and 1 due to paralysis of the affected upper extremity. Hence, 27 patients were included in this study.

The mean age at the time of surgery was 61.9±14.5 years; 22 were women and 5 were men. The mean time from injury to surgery was 3.7±2.9 days. The fracture position was the proximal third in 18 patients, the middle third in 9 patients, and the distal third in none. The fracture types according to the Arbeitsgemeinschaft für Osteosynthesefragen classification were: type A in 22 patients; type B in 4 patients; and type C in 1 patient.

Post-operatively, there were no cases of iatrogenic nerve injuries. Bone union was achieved within two years in all patients. The mean follow-up period was 7.5±2.1 years. Ultrasound examination at the final follow-up showed partial supraspinatus tear in 5 patients (18.5%) and complete tear in 5 patients (18.5%). There were no significant differences in patient demographic factors between patients without rotator cuff tears and those with partial or complete tears (Table I).

**Table I:** Comparison of patient characteristics by occurrence of rotator cuff tear.

	No tear (N=17)	Partial or complete tear (N=10)	P-value
Age * (years)	59.2±16.3	66.7±8.9	0.44
Sex †			0.62
Female	13	9	
Male	4	1	
Affected side of arm †			>0.99
Dominant arm	8	5	
Non-dominant arm	9	5	
Smoking †			>0.99
Yes	3	1	
No	14	9	
Diabetes †			>0.99
Yes	2	1	
No	15	9	
BMI * (kg/m^2^)	24.3±5.3	22.3±7.4	0.39
Time from injury to surgery * (days)	3.4±2.2	4.4±3.7	0.33
Time from surgery to final follow up * (years)	7.3±2.3	7.7±1.9	0.86
Pre-operative radial nerve injury †			>0.99
Yes	1	0	
No	16	10	
Position in shaft †			0.41
Proximal	10	8	
Middle	7	2	
AO classification †			0.12
A	13	9	
B	4	0	
C	0	1	

Notes - * Values are presented as means and standard deviations. † Values are presented as the number of patients. BMI: body mass index, AO: Arbeitsgemeinschaft für Osteosynthesefragen.

The mean adjusted Constant score, the ASES shoulder score, and the VAS score at the final follow-up were 86.4±12.7, 85.4±11.5, and 0.8±1.1, respectively. There were no significant differences in the adjusted Constant score, the ASES shoulder score, the VAS score, or range of shoulder motion at the final follow-up between patients without rotator cuff tears and those with partial or complete tears (Table II). In addition, there were no significant differences in the functional outcomes or incidence of rotator cuff tears among the three intramedullary nails used in this study (Supplementary Table I).

**Table II:** Comparison of clinical outcome scores and range of shoulder motion by occurrence of rotator cuff tear.

	No tear (N=17)	Partial or complete tear (N=10)	P-value
Adjusted Constant score	87.9±9.2	83.8±16.6	0.86
ASES shoulder score	86.7±9.9	83.1±13.6	0.64
VAS score	0.74±1.16	0.85±1.07	0.82
Range of shoulder motion			
Anterior elevation (°)	140±15	136±20	0.64
External rotation at sides (°)	46±14	43±14	0.57

Notes - Values are presented as means and standard deviations. ASES: American shoulder and elbow surgeons, VAS: visual analogue scale

**Fig. 2:**
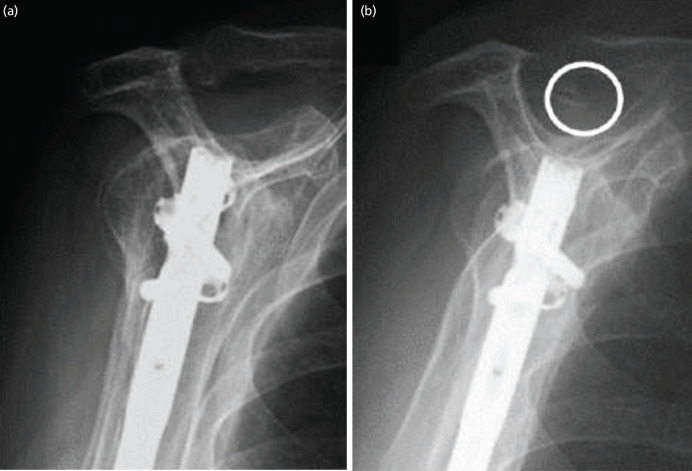
Plain radiographs of the right shoulder of a 79-year-old woman who underwent antegrade intramedullary nail fixation for humeral shaft fracture. (a) One-year after, and (b) five years after surgery. These radiographs show formation of the acromial spur at five years post-operatively.

Plain radiographs at the final follow-up showed glenohumeral osteoarthritis in no patients, acromial spurs in 8 patients (29.6%) (Fig. 2), and superior migration of the humeral head in 2 patients (7.4%). The incidence of acromial spurs was significantly higher in patients with partial or complete tears than in those without rotator cuff tears (70.0% vs. 5.9%, P<0.001) (Table III).

**Table III:** Comparison of radiographic outcomes by occurrence of rotator cuff tear.

	No tear (N = 17)	Partial or complete tear (N = 10)	P-value
Humeral head or glenoid rim spur	0	0	>0.99
Acromial spur	1	7	<0.001*
Superior migration of humeral head	0	2	0.13

Notes - Values are presented as the number of patients. * P<0.05

## Discussion

In this study, we investigated the incidence of rotator cuff tears at least five years after intramedullary nail fixation for humeral shaft fractures and the effect on mid-term shoulder functional and radiological outcomes. As a result, we made three important clinical observations.

First, this study demonstrated that 37% (10/27) of patients who underwent intramedullary nailing for humeral shaft fractures had partial or complete supraspinatus tendon tears at an average of 7.5 years after surgery. Previous studies of rotator cuff tears after intramedullary humeral nailing reported a 9–13% frequency of supraspinatus tendon tears 8–33 months post-operatively^11,12^, observed using ultrasonography. However, the incidence of post-operative rotator cuff tears in this study was higher than in these reports; this may partly be explained by the differences in post-operative follow-up times. One possible mechanism of rotator cuff tears after intramedullary nailing is that poor healing of the rotator cuff, with damage to the critical hypovascular region during nail insertion, leads to a long-term tear^4,6^. Our results suggest that the incidence of supraspinatus tendon tears after antegrade intramedullary nailing increases over time.

Second, our results revealed that post-operative rotator cuff tears had no significant effect on mid-term shoulder functional outcomes. Previous reports examining the association between rotator cuff tears after intramedullary nailing and short-term functional outcomes reported no significant association between the two factors^11,12^, which is congruent with the results of our study. However, recent studies on the outcomes of rotator cuff repair showed that post-operative re-tear does not affect short-term clinical outcomes, but worsens functional outcomes in the long-term, more than 10 years after surgery^16,17,26,27^. Given these findings, further long-term follow-up studies will be needed.

Third, patients with post-operative rotator cuff tears had a significantly higher incidence of post-operative acromial spurs on plain radiographs. Although it has been reported that secondary glenohumeral osteoarthritis occurs in 4.3% of patients after plate fixation of proximal humeral fractures^28^, there have been no studies that have radiologically evaluated the glenohumeral joint after intramedullary nailing for humeral shaft fractures. A previous study examining the radiographic outcomes of rotator cuff repair reported that acromial spur reformation five years post-operatively was significantly associated with the presence of re-tears^18^, a finding similar to the results of this study. Although acromial spurs have been associated with the presence of rotator cuff tears^22,29-33^, it is controversial whether they are a cause or consequence. This is also a limitation of our study, because no longitudinal ultrasonographic or radiographic evaluation was performed.

The main strength of this study is the evaluation of mid-term post-operative functional and radiological outcomes. Previous studies evaluating rotator cuff tears after humeral shaft fracture have evaluated outcomes 8–33 months postoperatively^11,12^, but no studies have evaluated ultrasonographic or radiographic outcomes more than 5 years post-operatively.

However, this study has certain limitations. First, because this was an observational study, biases from unobserved differences may have affected the results. For instance, although five surgeons performed the procedures, their skills were not taken into consideration. Nail design may influence post-operative outcomes. Two of the intramedullary nails (MultiLoc, Trigen) used in this study were straight nails inserted through the apex of the humeral head, while one (Polarus 2) was a lateral curved nail inserted from 4° lateral to the apex of the humeral head. However, the results of the subanalysis showed no significant differences in the functional outcomes or incidence of rotator cuff tears among the three nails used in the study. Second, in the present study, 14 patients were excluded due to loss to follow-up or refusal of radiographic examination, which may have reduced the generalisability of the post-operative results. Third, the small sample size is a major limitation of this study. For instance, a difference of 15 points in the Constant score was considered to be the minimal clinically important difference^34^. With a power of 80% and an α of 0.05, the power analysis demonstrated a sample size of 28 patients per group was needed; therefore, our results of functional outcome may have been affected by β-errors. Fourth, ultrasound examination was used for rotator cuff integrity in this study, which may be less valid and objective than magnetic resonance imaging.

## Conclusion

This study provides new information on rotator cuff tears following antegrade intramedullary nail fixation for humeral shaft fracture and its associated effects on mid-term functional and radiological outcomes. Our results revealed that 37% of patients developed partial or complete supraspinatus tendon tears at a mean follow-up of 7.5 years after surgery. Post-operative rotator cuff tears were significantly associated with the formation of acromial spurs; however, no significant effects on mid-term shoulder functional outcomes were noted.
